# New insights into the impact of *Lactobacillus* population on host-bacteria metabolic interplay

**DOI:** 10.18632/oncotarget.5906

**Published:** 2015-10-02

**Authors:** Caroline I. Le Roy, Jelena Štšepetova, Epp Sepp, Epp Songisepp, Sandrine P. Claus, Marika Mikelsaar

**Affiliations:** ^1^ Department of Food and Nutritional Sciences, The University of Reading, Whiteknights, Reading, United Kingdom; ^2^ Department of Microbiology, University of Tartu, Tartu, Estonia; ^3^ Bio-competence Center of Healthy Dairy Production LLC, Tartu, Estonia

**Keywords:** Lactobacillus, immunology, elderly, nuclear magnetic resonance, Gerotarget

## Abstract

We aimed at evaluating the association between intestinal *Lactobacillus* sp. composition and their metabolic activity with the host metabolism in adult and elderly individuals. Faecal and plasma metabolites were measured and correlated to the *Lactobacillus* species distribution in healthy Estonian cohorts of adult (*n* = 16; < 48 y) and elderly (*n* = 33; > 65 y). Total cholesterol, LDL, C-reactive protein and glycated hemoglobin were statistically higher in elderly, while platelets, white blood cells and urinary creatinine were higher in adults. Aging was associated with the presence of *L. paracasei* and *L. plantarum* and the absence of *L. salivarius* and *L. helveticus*. High levels of intestinal *Lactobacillus* sp. were positively associated with increased concentrations of faecal short chain fatty acids, lactate and essential amino acids. In adults, high red blood cell distribution width was positively associated with presence of *L. helveticus* and absence of *L. ruminis*. *L. helveticus* was correlated to lactate and butyrate in faecal waters. This indicates a strong relationship between the composition of the gut *Lactobacillus* sp. and host metabolism. Our results confirm that aging is associated with modulations of blood biomarkers and intestinal *Lactobacillus* species composition. We identified specific *Lactobacillus* contributions to gut metabolic environment and related those to blood biomarkers. Such associations may prove useful to decipher the biological mechanisms underlying host-gut microbial metabolic interactions in an ageing population.

## INTRODUCTION

The human intestinal tract harbours a large and complex community of microorganisms [1] that varies widely across the population. Studies report more than 1,000 different species of bacteria present in the large intestine of healthy adults depending on the technic used to asses this number [1-5]. The complex composition of the gut microbiota is now recognized to be strongly involved in host metabolic and immunologic homeostasis [6, 7]. This impact on host health status is partially due to the important metabolic role played by the gut microbial ecosystem during digestion [8]. This symbiotic balance is extremely complex to understand due to a multitude of factors affecting its modulation, such as age, diet and lifestyle [9, 10]. Numerous publications have explored the impact of specific phyla and genera on host health. For example, higher *Bacteroidetes*/*Firmicutes* ratio has been associated with lean constitution [11-13] in rodents and lower plasma glucose concentration in diabetic humans [14].

*Lactobacillus* are part of the Firmicutes genus, to date over hundred species have been described of which 30% are found in the human gastro intestinal (GI) track [15]. *Lactobacillus* species are commonly used as probiotics because of their ability to protect the host against pathogen invasion, improve intestinal barrier function, provide metabolic and immunologic health-promoting properties [16]. However, contradictory results have been published regarding the possible association of some *Lactobacillus* species with host metabolic homeostasis. For example, although *L. casei* and *L. gasseri* have been often associated with beneficial metabolic outcomes against obesity and diabetes [17, 18] some studies have shown a positive association between a higher diversity of *Lactobacillus* sp. and increased BMI and glycaemia in humans [19]. In addition, the genome characterization of some *Lactobacillus* sp. strongly associated with high BMI has revealed a lack of enzymes involved in carbohydrate metabolism [20-22]. Moreover, it has been shown that within this genus, some *Lactobacillus* probiotic strains, such as *Lactobacillus acidophilus*, *L. casei*, and *L. rhamnosus*, induced differential gene-regulatory transcriptional networks and pathways in the human mucosa [22]. In another study, *L. reuteri* was found predominantly associated with obese adults compared to lean subjects [23], raising questions about the overall impact of some of these *Lactobacillus* species on the host metabolic homeostasis. As a consequence, a recently published meta-analysis on the impact of specific *Lactobacillus* species on weight gain [24] highlighted the urgency to explore the specific metabolic impact of species and even strains on the host. This further supports that every *Lactobacillus* probiotic strain triggers a specific host response that must be considered for a more personalised approach to functional food [25].

Elderly ( > 65 years) are the fastest growing subpopulation in the world. It has been shown that aging increased the viable count of *Lactobacillus* with substantial changes in species prevalence [26-28]. Coincidently, the high inter-individual variability of the *Lactobacillus* species distribution is enhanced by age variations [19]. Elderly experience a profound modification in the composition of their gut microbiota [29], which has been associated in several studies with loss of immune functions and increased cardiovascular disease risk. Yet, it is not clear how the *Lactobacillus* genus impacts host metabolism. Thus, assessing the potential implication of the *Lactobacillus* population composition and its metabolic activity on physiological modifications occurring with aging is important for future corrections of microbial dysbiosis associated with age. Moreover, there is a lack of information about the metabolic activity of the gut microbial ecosystem in relation with *Lactobacillus* sp. composition. ^1^H NMR-based metabonomics is a powerful approach to identify the missing relationships between the gut microbial composition, gut metabolites and the host health [30-32]. To date, only a few publications have addressed the impact of specific *Lactobacillus* species on the metabolic profile of rat biofluids and gut tissues [33].

In this study we aimed at evaluating the impact of intestinal *Lactobacillus* sp. composition on several blood biomarkers of metabolic homeostasis that may be altered with age. To evaluate such association, fecal samples collected from two cohorts of healthy adult (*n* = 16) and elderly individuals (*n* = 33) recruited in previous studies [19] were analysed using a metabonomics approach. We used volunteers that formed a cohort of elderly individuals where the impact of a profile of *Lactobacillus* sp. on anthropometric parameters (white blood cell count and oxidized LDL) had been investigated [34]. We also used volunteers derived from a cohort of healthy adults and elderly where we questioned the impact of the *Lactobacillus* profile on BMI and glycaemia [19]. In this work, we present unexplored associations between the population of *Lactobacillus* and host metabolism in these two age groups using a metabonomics approach.

## RESULTS

### Association between physiological modifications of blood biomarkers of homeostasis with aging

**Figure 1:**
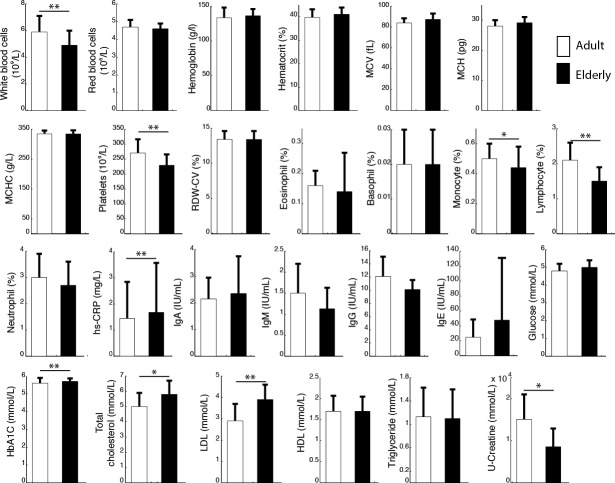
The figure represents the average measurement of several blood biomarkers for adults (open bars) and elderly (close bars) Results are presented as mean ± SD; **p < 0.01; *p < 0.05. Key: MCV, mean corpuscular volume; MCH, mean corpuscular hemoglobin; MCHC, mean corpuscular hemoglobin concentration; RDW-CV, red cell distribution width; Hs-CRV, high sensitivity C-reactive protein; HbA1C, glycated hemoglobin; LDL, low density lipoprotein; HDL, high density lipoprotein.

Clinical indices reflecting the general homeostasis of all participants in both groups are summarized in Figure 1. Levels of total cholesterol, low-density lipoproteins (LDL), high sensitivity C-reactive protein (hs-CRP) and glycated hemoglobin (HbA1C) were significantly higher in elderly compared with adults (*p* = 0.03; *p* = 0.001; *p* < 0.001; *p* = 0.002, respectively). To the contrary, platelets, white blood cell count and urinary creatine (U-Creatine) were higher in adults (*p* = 0.002; *p* = 0.007; *p* = 0.026, respectively).

**Figure 2:**
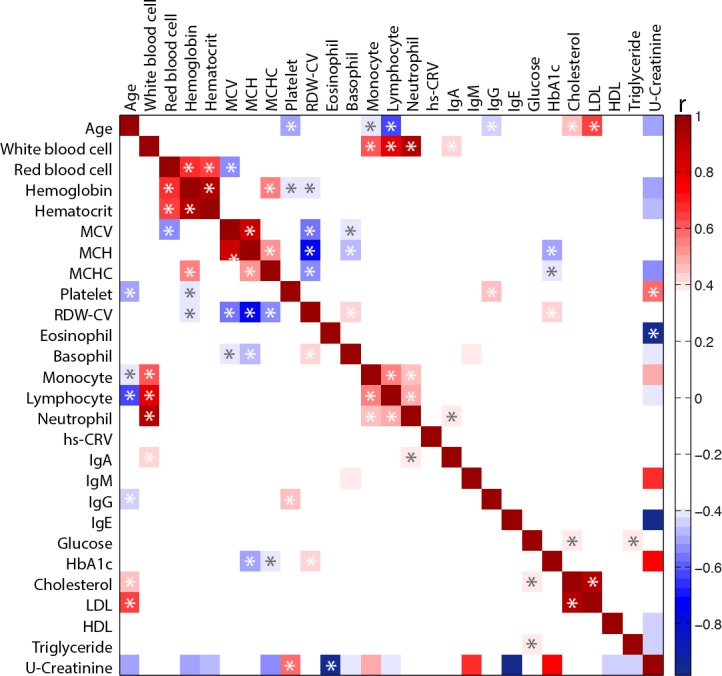
Heat map of the Spearman correlation (*r*) between blood biomarkers Correlations were performed between each blood biomarker for the overall population. Key: MCV, mean corpuscular volume; MCH, mean corpuscular hemoglobin; MCHC, mean corpuscular hemoglobin concentration; RDW-CV, red cell distribution width; Hs-CRV, high sensitivity C-reactive protein; HbA1C, glycated hemoglobin; LDL, low density lipoprotein; HDL, high density lipoprotein. *Significant correlation (*p* < 0.05) after correction using Benjamini and Hochberg false discovery rate method with a false discovery rate Q = 0.20.

In order to identify potentially hidden relationships between blood markers presented in Figure 1, a Spearman correlation coefficient (*r*) was calculated between each blood biomarker independently and with age (Figure 2). As expected, blood biomarkers related to immunity and inflammation were clustered together. Similarly, hemoglobin parameters (i.e. red blood cell, hematocrit and hemoglobin) formed a distinct cluster. As observed in Figure 1, age was negatively correlated with platelet, monocyte, lymphocyte and IgG but positively correlated with total cholesterol and LDL levels. Interestingly, a negative correlation between RDW-CV and corpuscular parameters (i.e. MCV, MCHC and MCH) was also observed.

### Gut microbial *Lactobacillus* sp. composition is associated with age and blood clinical indices

**Figure 3:**
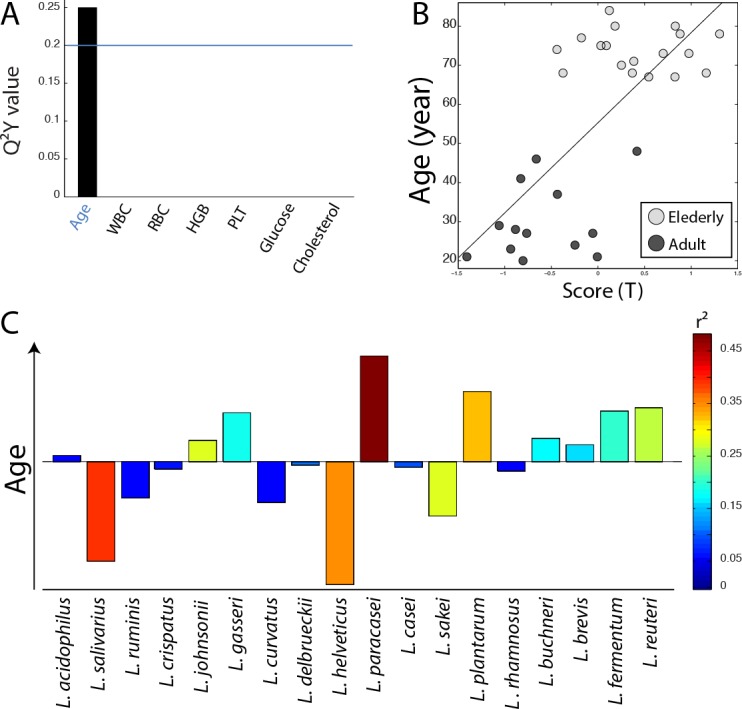
Association of *Lactobacillus* species profile with physiological parameters **A.** Histogram of descending Q^2^Y values derived from O-PLS models performed using the profile of *Lactobacillus* species as X matrix and the quantitative levels of 7 physiological parameters as independent response predictors for the overall population (*N* = 32). **B.** Scores derived from the O-PLS model using age as a response predictor (R^2^Y = 0.51, R^2^X = 0.16, Q^2^ = 0.25, *p*-value = 0.003 and *p*-value FDR = 0.006). **C.** Loadings plot derived from the same model as in **B.**; *Lactobacillus* species pointing upward on the loadings plot are positively correlated with the elderly group, and *Lactobacillus* species pointing downward are correlated with the adult population.

**Figure 4:**
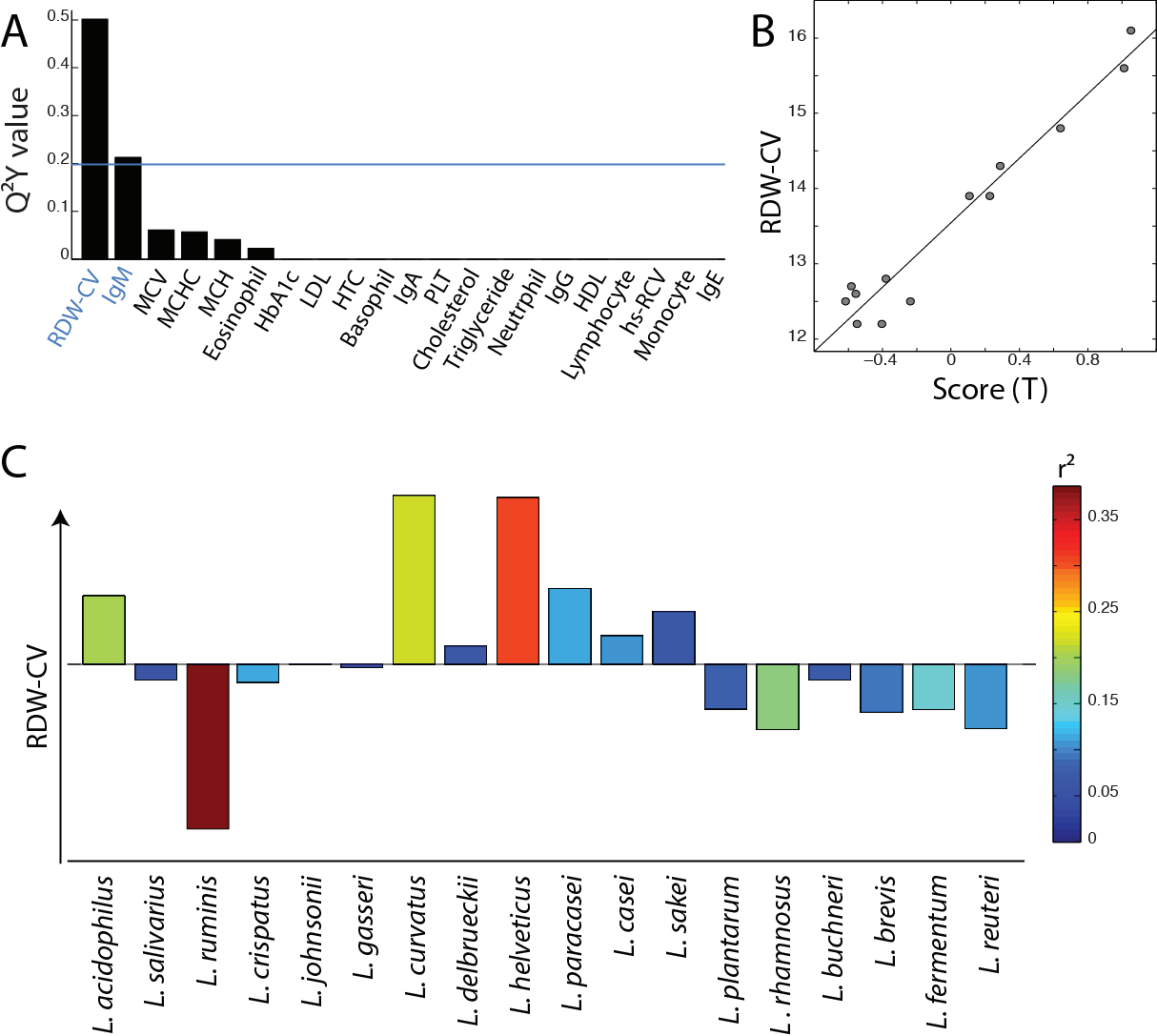
Association of *Lactobacillus* species profile with blood biomarkers in adult individuals **A.** Histogram of descending Q^2^ values calculated from O-PLS model performed using the profile of *Lactobacillus* species as X matrix and the quantitative levels of 21 blood biomarkers as independent response predictors for the adult population only. **B.** Scores derived from the O-PLS model using RDW levels as a response predictor (this model uses one orthogonal component; R^2^Y = 0.95, R^2^X = 0.28, Q^2^Y = 0.67, *p*-value = 0.005 and *p*-value FDR = 0.013). **C.** Loadings plot derived from the same model as in **B.**, *Lactobacillus* species pointing upward on the loadings plot are positively correlated with RDW levels.

In order to evaluate the potential implication of the gut *Lactobacillus* sp. composition on health, the association between the profile of *Lactobacillus* species and twenty-six physiological parameters described in Figure 1 (including age, immunological indices and plasma biochemical biomarkers) was evaluated using O-PLS regressions. Since some parameters were not available for the overall cohort, some associations were either evaluated for both age groups together (Figure 3) or separately (Figure 4), as appropriate. Models were selected for further investigations when the Q^2^Y value was greater than an arbitrary threshold of 0.20 as presented in Figures 3A and 4A. Based on these criteria, models fitting red cell distribution width (RDW) levels, age and IgM concentrations were selected. Validations of these models were obtained by random permutation test (see material and methods). As a result, the IgM model was further rejected due to a *p*-value exceeding the risk alpha threshold of 5% (*p*-value = 0.062). As previously described, aging was associated with the presence of *L. paracasei* (r^2^ = 0.48) and *L. plantarum* (r^2^ = 0.30) and the absence of *L. salivarius* (r^2^ = 0.37) and *L. helveticus* (r^2^ = 0.34) in this cohort (Figure 3C). This new analysis revealed that high RDW levels were positively associated with the presence of *L. helveticus* (r^2^ = 0.29) and the absence of *L. ruminis* (r^2^ = 0.36) (Figure 4C).

### Total population of *Lactobacillus* impacts faecal metabolic profiles

**Figure 5:**
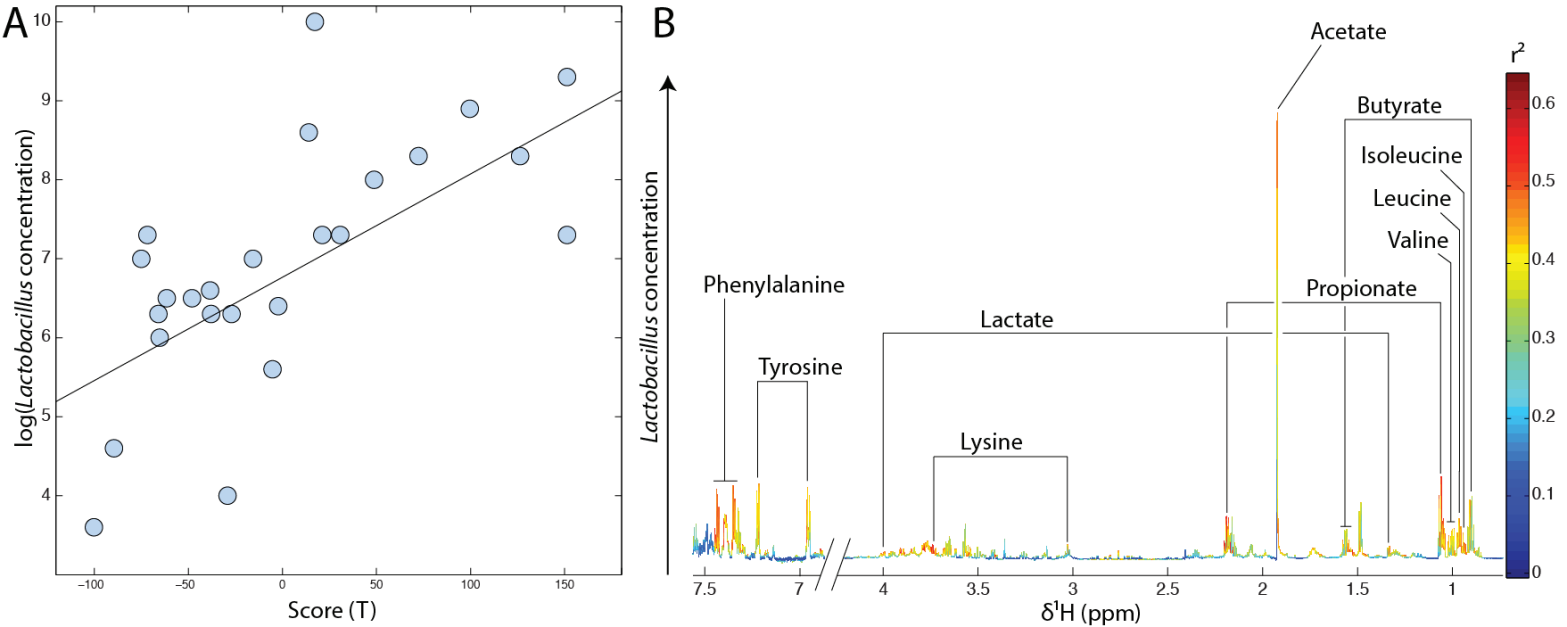
High total count of *Lactobacillus sp*. is associated with higher faecal SCFAs, lactic acid and amino acid levels **A.** O-PLS scores projection of all individuals according to the decimal logarithm of total *Lactobacillus* counts. This model is derived from ^1^H-NMR spectra of faecal waters using the log of total *Lactobacillus* counts as a response predictor. **B.** Metabolic contribution of SCFAs, organic acids and amino acids to the same model (loadings plot). Metabolites pointing upwards are positively correlated with high *Lactobacillus* levels.

Faecal metabolic profiles reflect the overall metabolic activity of the gut microbial ecosystem. In order to get insights into the contribution of specific bacterial species to the gut metabolic environment, we used a series of linear regressions using O-PLS algorithms. Hence, impact of total *Lactobacillus* count on the faecal metabolic profile was evaluated by O-PLS regression using the decimal logarithm of *Lactobacillus* concentration as a response predictor. Results displayed in Figure 5 reveal a strong correlation (R^2^Y = 0.44, Q^2^ = 0.27 and *p*-value = 0.002) between the *Lactobacillus* counts found in faeces and individual's faecal metabolic profiles. High levels of *Lactobacillus* sp. were positively correlated with increased SCFAs (acetate, propionate and butyrate), lactic acid and essential amino acid (tyrosine, phenylalanine, leucine, isoleucine, valine and lysine) levels (Figure 5B).

### *L. helveticus* shows specific contributions to fecal metabolic profiles

**Figure 6:**
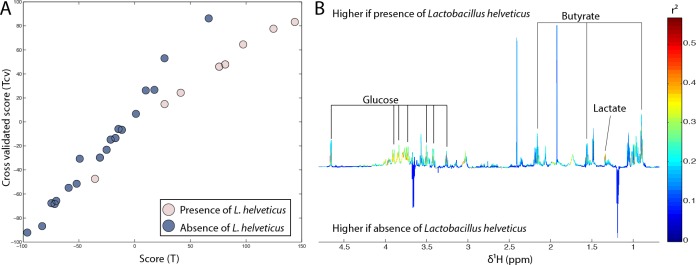
Presence of *L. helveticus* was associated with an increase in butyrate and lactate faecal counts **A.** Scores plot derived from the O-PLS model using the absence (dark blue) or presence (light pink) of *L. helveticus* as a response predictor. The calculated scores (x axis) are plotted against the cross-validated scores (y axis). **B.** Loadings plot derived from the same model showing the metabolic contribution of butyrate, lactate and glucose. Metabolites pointing upwards correlate with the presence of *L. helveticus*.

In order to determine the individual impact of the presence of sixteen *Lactobacillus* species on faecal metabolic profiles regardless of age, O-PLS models were performed using bacterial presence or absence as a response predictor (presence was coded 1 and absence was coded 0 in the response vector). Only the presence of *L. helveticus* appeared to significantly influence faecal metabolic profiles in this cohort (R^2^Y = 0.50, Q^2^Y = 0.27, *p*-value = 0.008) (Figure 6). Butyrate, lactate and glucose levels were positively correlated with the presence of *L. helveticus*.

## DISCUSSION

Worldwide, the increased percentage of elderly in the population has steered attention towards a better understanding of physiological changes accompanying aging. Among them, decreased digestion, immune function [35, 36] and increased risk of developing metabolic syndrome during senescence are of major importance. In this context, understanding the regulatory mechanisms triggering such changes will significantly help to improve elderly's quality of life. As there is increasing evidence that the gut microbiota, and more specifically the *Lactobacillus* genus, may be a key player in these transformations [29, 37], we analysed various blood biomarkers of homeostasis and quantified the profile of *Lactobacillus* sp. in two age groups. Although both groups were considered healthy, elderly presented lower counts of white blood cells, especially lymphocytes and monocytes (Figure 1). Our results also showed that blood metabolic parameters in elderly differed from the adults because of higher cholesterol content (total cholesterol and LDL), and higher insulin resistance (HbA1c) (Figure 1). These results are consistent with previously assessed immunologic and metabolic variations related to aging [38, 40], which supports the use of this cohort to evaluate associations between these biomarkers and the profile of gut *Lactobacillus*.

We further looked for associations between the total count and the composition of intestinal lactobacilli species with age, BMI and every blood biomarker presented in Figure 1. These results were in accordance with previously described data where aging was characterized by a modification of the *Lactobacillus* population [41, 42]. *L. paracasei* and *L. plantarum* were predominantly found in elderly whereas *L. salivarius* and *L. helveticus* were in higher levels in adults. We did not observe here that *L. acidophilus* was more predominantly found in adults than in elderly as previously published, which is likely a consequence of the use of smaller subgroups that may have impacted the statistical power of the present analysis [19]. In addition, it is possible that variations in diet may be responsible for inconsistent results between studies. Nevertheless, since modifications of the gut microbiota over a lifespan have been documented worldwide, it is likely that other factors are involved. None of the immunologic and metabolic biomarkers differentiating elderly from adults (i.e. total cholesterol, LDL, white blood cells, lymphocyte, monocyte, U-creatinine, hs-CRP, platelet, and glycated hemoglobin) generated robust O-PLS models when associated with the species profile of *Lactobacillus*. The work presented here was a focus on the influence of *Lactobacillus* species on the host metabolism and therefore we did not perform a deep sequencing analysis of the microbial ecosystem that would have been necessary to identify co-varying microbes that may contribute to a potential impact of the whole *Lactobacillus* genus on the host metabolism.

Interestingly, a strong model was found when the red cell distribution width (RDW-CV) was used as a response predictor for the *Lactobacillus* sp. composition in the adult population. This blood biomarker was positively associated with the presence of *L. helveticus* and the absence of *L. ruminis*. No significant variation of the RDW-CV was found between the two age groups, indicating that this result was not directly related to ageing. The three other red blood cell biomarkers: mean corpuscular volume (MCV), mean corpuscular hemoglobin concentration (MCHC) and mean corpuscular hemoglobin (MCH) were negatively associated with RDW-CV and are often measured as indicators of anemia [43]. High RDW-CV and low MCV are generally associated with microcytic anemia [44]. This type of anemia can be caused by iron, vitamin B_12_ and/or folic acid deficiency in the bloodstream [45-47]. Such deficiency usually results from diet depletion and/or poor absorption by the gut barrier of these methyl donors. Interestingly, the presence of *L. helveticus*, a bacterium mostly found in adults that is not able to synthetize vitamin B_12,_ was positively associated with RDW-CV [48]. It has been shown that this *Lactobacillus* species expresses a receptor for vitamin B_12_ [49] resulting in significant depletion of the vitamin B_12_ in milk during fermentation [50]. Therefore the presence of *L. helveticus* in the gut might significantly decrease the bioavailability of vitamin B_12_ for the host resulting in increased risks of anemia.

Concurrently, we observed a negative correlation between the presence of *L. ruminis* and RDW-CV. Although the sequenced genome of *L. ruminis* showed its inability to produce vitamin B_12_ [51], a recent publication investigating the potential of several lactobacilli as vitamin producers demonstrated that *L. ruminis* possessed all the genes necessary for chorismate biosynthesis [52]. Chorismate is a precursor of folic acid, the insufficiency of which is also linked to microcytic anemia. Since *L. ruminis* appears to lack various genes necessary to complete folate biosynthesis, it can therefore be hypothesized that *L. ruminis* secrets chorismate in the gut environment, which is then used as a substrate by other commensal bacteria for folic acid production that may be made available to the host [53]. This hypothesis will have to be further tested to validate the potential opposite roles of *L. helveticus* and *L. ruminis* in vitamin B_12_ bioavailability.

NMR-based metabonomics was used to measure all detectable low molecular weight metabolites in fecal waters as a reflection of the metabolic activity of the gut microbial ecosystem. This approach has proved particularly helpful to detect relationships between the gut microbial composition and the host metabolism [30, 32]. To date only a few publications have addressed the impact of specific *Lactobacillus* species (*L. acidophilus* and *L. paracasei*) on the metabolic profile of rat biofluids and gut tissues [33]. Here, we found no correlation between faecal metabolic profiles and blood biomarkers, indicating that the overall metabolic activity of the gut microbial ecosystem may not be directly associated with modifications of blood biochemistry. Nonetheless, it was possible to identify significant associations between the gut profile of *Lactobacillus* sp. and faecal water metabolic profiles. Higher counts of total lactobacilli were associated with a higher content in essential amino acids (tyrosine, phenylalanine, leucine, isoleucine, valine and lysine) and SCFA (butyrate, propionate and acetate) and lactate. This is consistent with the diversity of metabolic pathways used by intestinal *Lactobacillus* sp. for degradation of a large range of metabolites including carbohydrates, lipids and proteins [54]. Because *Lactobacillus* sp. are not the main SCFAs producers, this suggests cross-feeding interactions with other SCFAs-producing bacteria and indicates that *Lactobacillus* sp. markedly contribute to the overall SCFAs content in the lumen. SCFAs production by the gut microbiota is of crucial importance because of their wide range of action on the host [55]. Indeed, SCFAs have been associated with a protective effect against colon cancer development [56, 57] and cardiovascular disease risk [58]. In this study, elderly presented higher *Lactobacillus* count than adults. However, ^1^H-NMR faecal metabolic profiles did not reveal any significant difference between adults and elderly, indicating that SCFAs concentrations were not significantly different between the two age groups despite the difference in total *Lactobacillus* count. This is not surprising since many bacterial species are able to produce SCFAs from the fermentation of carbohydrates and proteins [59], and the increase in total *Lactobacillus* in elderly may not be sufficient to increase the overall counts of SFCAs in faecal waters. This observation is in contradiction with previous studies, which demonstrated that age was associated with an overall reduction in faecal excretion of SCFAs related to decreased bacterial diversity [60, 61].

Regardless of age, *L. helveticus* were strongly associated with variations in faecal metabolic profiles. The presence of *L.helveticus* was associated with increased butyrate and lactate levels in faecal waters, indicating the significant metabolic contribution of *L. helveticus* to the gut metabolic environment. *L. helveticus* is a strictly homofermentative bacteria that can only produce lactate from the fermentation of saccharides, and is not a butyrate producer. Therefore, the correlation with butyrate is indicative of bacterial cross-feeding where lactate produced by *L. helveticus* may be used by butyrate-producing bacteria. Such co-metabolism has been demonstrated in the human gut where several bacteria including *Eubacterium hallii* and *Anaerostipes caccae* use lactate to produce butyrate [62].

In conclusion, this study provides new insights into the complex metabolic network existing between the host and its gut microbiota. The global composition of *Lactobacillus sp*. significantly impacts the metabolic environment of the gut, promoting the total amount of lactate, SFCAs and essential amino acids. We also highlighted the unique metabolic contribution of specific *Lactobacillus* species to the host-gut microbial metabolic interactions. We therefore confirm that a bacterial genus can play an important role in host health and demonstrate that specific metabolic contributions of independent species should also be evaluated. This emphasizes the need for a deeper understanding of the host-gut bacterial metabolic interactions that must pave the way for a personalized approach to probiotic supplementation.

## MATERIALS AND METHODS

### Study population

We used faecal samples, blood biomarkers and *Lactobacillus* profiles of two healthy Estonian cohorts of adult (*n* = 16; 20 48 y) and elderly (*n* = 33; 65-81 y) individuals. The experimental design applied to these cohorts was approved by the Ethics Committee of the Medical Faculty of the University of Tartu with approvals no. 139/16 20.06.2005, no. 158/10, 26.03.2007 and no. 184/T-10, 26.08.2009. The present study was conducted according to the guidelines laid down in the Declaration of Helsinki.

All 16 adults and 17 of the elderly subjects were recruited from the trials ISRCTN38739209 and ISRCTN15061552, respectively, assessing the impact of a probiotic provided by Bio-competence Centre of Healthy Dairy Products LLC, as previously described [19]. For the purpose of this publication, we only analysed the data of baseline at recruitment pre-intervention. The additional sixteen elderly were selected from the registry of family doctors and orthopedists of the Tartu University Hospital, Estonia, before performing elective orthopedic surgery, according to inclusion and exclusion criteria as presented in the original publications [19, 34]. The selection of the final cohort (*N* = 49) of individuals included in this study was based on the availability of frozen faecal samples to perform ^1^H-NMR metabolic profiling. All individuals in both age groups were healthy and followed a standard Western-type diet, typically rich in potatoes, vegetables, meat, eggs but characterised also by a high content of fibre (rye bread, oat/wheat/rice porridge) and dairy products, vegetable seed oils, margarine and non-alcoholic beverages.

Faecal *Lactobacillus* species distribution assessed by real-time quantitative PCR have been previously published by Stsepetova et al. [19]. These data were used in order to evaluate potential association between the *Lactobacillus* sp. composition and several blood biomarkers and faecal metabolic profiles. Some of the blood clinical indices have been published previously for the elderly and adult cohort but not compared to each other and put in relationship with the *Lactobacillus* sp. composition and its metabolic activity [34, 63].

### Sample collection and preparation

Fresh stool samples were placed into sterile containers. The samples collected at home or at hospital were kept in a domestic refrigerator at 4°C for no more than 2h before transportation to the laboratory. Upon arrival, samples were mechanically homogenized with a sterile spatula, divided into aliquots and stored at −70°C until future DNA isolation and molecular microbiological analysis.

### Molecular analysis of *Lactobacillus* sp. in fecal samples

Methods for quantitative and qualitative molecular analysis of *Lactobacillu*s sp. in feces have been described in a previous study [19]. DNA was extracted from stool samples using a QIAamp DNA stool mini kit (Qiagen N.V.) with some modifications. 1g of feces was suspended in 10 mL of PBS buffer and homogenized. 0.3 g of 0.1 mm zirconia/silica beads and 1.4 mL of ASL solution from the stool mini kit was added to 1 mL of pellet cells. The tubes were then agitated for 3 minutes at a speed of 5000 rpm in a mini-bead beater (Biospec Products Inc.). The protocol was then continued as described by the manufacturer (Qiagen N.V.).

In order to establish a quantitative assay, we cloned plasmids containing the amplified region of the target bacteria *L. paracasei* using the pGEM-T vector system (Promega, Madison, WI, USA) [64]. Quantification of target DNA was achieved by using serial tenfold dilution from 10^2^ to 10^9^ plasmid copies of the previously quantified plasmid standards. Real-time PCR was performed with the ABI PRISM 7500 HT Sequence Detection System (Applied Biosystems, Bedford, MA, USA) using optical-grade ninety-six-well plates. The PCR was performed on a total volume of 25 μL using SYBR Green PCR Master mix (Applied Biosystems). Each reaction included 150 ng of template DNA, 12.5 μL of SYBR Green PCR Master mix (Applied Biosystems) and 2 mM of each primer [65]. The conditions were set as follows: 2 min at 50°C and 10 min at 95°C, followed by forty cycles consisting of denaturation at 95°C for 15 s, and annealing and elongation at 60°C for 1 min. Data analysis was conducted with Sequence Detection Software version 1.6.3, supplied by Applied Biosystems.

The *Lactobacillus* species-specific qualitative PCR was carried out by primers targeted on the 16S 23S ribosomal RNA intergenic spacer region [66]. A reaction mixture (50 μL) consisted of 10X reaction buffer, a 200 μM concentration of each deoxynucleoside triphosphate, 1 μM of each primer, 100 ng of bacterial DNA (extracted from the faecal samples) and 1.5 U of HotStar Taq Plus DNA polymerase (Qiagen). The amplification program consisted of predenaturation at 94°C for 5 min, followed by thirty-five cycles of 94°C for 30 s, 30 s at the appropriate annealing temperature [19], and finally 72°C for 30 s. A cycle of 72°C for 10 min concluded the program.

### Blood biochemistry

Blood samples were obtained in the early morning after 8 h of fasting. Samples were drawn from the antecubital vein with a vacutainer into heparinised tubes and immediately stored (on ice) at 4°C. Data of hematological indices: hemoglobin, red blood cells, leukocytes, lymphocytes, monocytes, basophil, eosinophil, neutrophil, platelets, red cell distribution width (RDW-CV), mean corpuscular volume (MCV), mean corpuscular hemoglobin concentration (MCHC), mean corpuscular hemoglobin (MCH), hematocrit (HCT); inflammatory indices: white blood cells count (WBC), high sensitivity C-reactive protein (hs-CRP); metabolic indices: plasma glucose, glycated hemoglobin (HbA1C), total cholesterol, oxidized LDL (ox-LDL), low density lipoproteins (LDL-C), high density lipoproteins (HDL-C) and triglycerides (TG); immunological: immunoglobulin E (IgE), immunoglobulin M (IgM), immunoglobulin G (IgG), immunoglobulin A (IgA); and urinary creatinine (U-creat) were performed with standard laboratory methods using certified assays in the clinical laboratory of the Tartu University Clinics, Estonia.

Intervals for routine laboratory tests proposed by the Nordic Reference Interval Project (NORIP, http://www.furst.no/norip/) were used as references.

### Extraction of polar metabolites

Faeces were homogenized in 1 mL of phosphate buffer (prepared in 9:1 D_2_O/H_2_O and 0.05 % sodium 3-(tri-methylsilyl)propionate-2,2,3,3-d_4_ (TSP) as a ^1^H NMR reference) for 5 min at T = 1/25 in a tissue lyser (TissueLyser LT, Qiagen). Samples were then centrifuged for 10 minutes at 22000 g. Supernatants were removed into new Eppendorf tubes and 500 μL of faecal water extract were transferred to 5mm NMR glass tubes for analysis.

### ^1^H-NMR -based metabonomics

^1^H NMR spectra of faecal waters were acquired on a Bruker Avance DRX 700 MHz NMR Spectrometer (Bruker Biopsin, Rheinstetten, Germany) operating at 700.19 MHz using a standard 1-dimensional (1D) pulse sequence [recycle delay (RD)-90°-*t1*-90°-*tm*-90°-acquire free induction decay (FID)] with water suppression applied during RD of 2 s and the mixing time (*tm*) of 100 ms and a 90 degree pulse set approximately at 10 μs. For each spectrum, a total of 256 scans were accumulated into 32 K data points with a spectral width of 12019 Hz. A range of 2D NMR spectra was performed on the same equipment on selected samples, including correlation spectroscopy (COSY) NMR spectroscopy. The FIDs were multiplied by an exponential function corresponding to 0.3 Hz line broadening. All spectra were manually phased, baseline corrected and calibrated to the chemical shift of TSP (δ 0.00) in MNova NMR version 8 (Mestrelab Research, Spain). Metabolites were assigned using 2D NMR experiments, data from literature [67] and our in house database of standards.

### Statistical analysis

Statistical analysis of blood biochemical markers was performed using R 3.0.2 (A Language and Environment, http://www.r-project.org) and GraphPad Prism version 4.00 for Windows (GraphPad Software, San Diego, CA). Clinical and biochemical data were expressed as mean ± standard. Adults and elderly were compared by *t*-tests or the Wilcoxon rank sum test according to the distribution of data adjusted for multiple comparisons. Differences were considered statistically significant if the *p*-value was strictly inferior to 0.05.

Spearman correlation coefficients were calculated between every blood markers independently and with age using Matlab software (The Mathworks, version R2013a). Statistical significance was evaluated using Benjamini and Hochberg false discovery rate correction (Q = 0.20, *n* = 31).

Prior to analysis, all ^1^H-NMR spectra were digitalized and imported into Matlab version R2013a. The residual water signal was removed between 4.70 and 5.10 ppm. All spectra were then mean centered and scaled to unit variance. Principal Component Analysis was used to observe the general variation in the dataset and detect potential outliers. Data were then analysed using orthogonal projection to latent structure (O-PLS) where ^1^H-NMR spectra were used as a matrix of independent variables (X) for modeling age, microbial composition and blood marker concentration as predictors (Y). The following parameters were considered for each O-PLS model: R^2^Y (goodness of fit: percentage of Y explained by the model) and Q^2^Y (the goodness of prediction: percentage of Y predicted after 7-fold cross validation). Significance of selected models was validated by 500 random permutation tests.

In order to identify specific metabolic associations with individual bacterial species, a similar statistical approach was adopted using O-PLS regression analysis where the *Lactobacillus* species composition was used as a matrix of independent variables (X) for modeling blood physiological parameters (Y). The same parameters as the one described for the O-PLS analysis using ^1^H-NMR spectra were considered for model selection. *p*-values obtained by random permutation tests were corrected using the Benjamini & Hockberg false discovery rate method (Q = 0.20).
